# Supplementary material for: COZOID: contact zone identifier for visual analysis of protein-protein interactions
Source: BMC Bioinformatics. 2018 Apr 6;19:125. doi: 10.1186/s12859-018-2113-6 (PMC5889581; doi:10.1186/s12859-018-2113-6)
Supplement: Supplementary file 2 — Software build. Executable binary file of the software tool. (ZIP 118,784 kb) [file 12859_2018_2113_MOESM2_ESM.zip › cozoid/java/docs/beansbinding-1.2.1-doc.zip!/org/jdesktop/el/impl/parser/Node.html]

Node (Beans Binding)


---


|  |  |  |
| --- | --- | --- |
| **Overview** \| **Package** \| **Class** \| **Use** \| **Tree** \| **Deprecated** \| **Index** \| **Help** | |  |
| **PREV CLASS**   **NEXT CLASS** | **FRAMES**    **NO FRAMES**     **All Classes** |  |
| SUMMARY: NESTED \| FIELD \| CONSTR \| METHOD | DETAIL: FIELD \| CONSTR \| METHOD |  |


---


## org.jdesktop.el.impl.parser Interface Node

**All Known Implementing Classes:**: ArithmeticNode, AstAnd, AstBracketSuffix, AstChoice, AstCompositeExpression, AstDeferredExpression, AstDiv, AstDotSuffix, AstDynamicExpression, AstEmpty, AstEqual, AstFalse, AstFloatingPoint, AstFunction, AstGreaterThan, AstGreaterThanEqual, AstIdentifier, AstInteger, AstLessThan, AstLessThanEqual, AstLiteralExpression, AstMinus, AstMod, AstMult, AstNegative, AstNot, AstNotEqual, AstNull, AstOr, AstPlus, AstString, AstTrue, AstValue, BooleanNode, SimpleNode

---

``` public interface Node ```

---

| **Method Summary** | |
| --- | --- |
| `void` | `accept(NodeVisitor visitor)` |
| `java.lang.String` | `getImage()` |
| `MethodInfo` | `getMethodInfo(EvaluationContext ctx, java.lang.Class[] paramTypes)` |
| `java.lang.Class` | `getType(EvaluationContext ctx)` |
| `java.lang.Object` | `getValue(EvaluationContext ctx)` |
| `java.lang.Object` | `invoke(EvaluationContext ctx, java.lang.Class[] paramTypes, java.lang.Object[] paramValues)` |
| `boolean` | `isReadOnly(EvaluationContext ctx)` |
| `void` | `jjtAddChild(Node n, int i)`             This method tells the node to add its argument to the node's list of children. |
| `void` | `jjtClose()`             This method is called after all the child nodes have been added. |
| `Node` | `jjtGetChild(int i)`             This method returns a child node. |
| `int` | `jjtGetNumChildren()`             Return the number of children the node has. |
| `Node` | `jjtGetParent()` |
| `void` | `jjtOpen()`             This method is called after the node has been made the current node. |
| `void` | `jjtSetParent(Node n)`             This pair of methods are used to inform the node of its parent. |
| `void` | `setValue(EvaluationContext ctx, java.lang.Object value)` |

**Method Detail**

### jjtOpen

```
void jjtOpen()
```

:   This method is called after the node has been made the current
    node. It indicates that child nodes can now be added to it.

---


### jjtClose

```
void jjtClose()
```

:   This method is called after all the child nodes have been
    added.

---


### jjtSetParent

```
void jjtSetParent(Node n)
```

:   This pair of methods are used to inform the node of its
    parent.

---


### jjtGetParent

```
Node jjtGetParent()
```

---


### jjtAddChild

```
void jjtAddChild(Node n,
                 int i)
```

:   This method tells the node to add its argument to the node's
    list of children.

---


### jjtGetChild

```
Node jjtGetChild(int i)
```

:   This method returns a child node. The children are numbered
    from zero, left to right.

---


### jjtGetNumChildren

```
int jjtGetNumChildren()
```

:   Return the number of children the node has.

---


### getImage

```
java.lang.String getImage()
```

---


### getValue

```
java.lang.Object getValue(EvaluationContext ctx)
                          throws ELException
```

:   **Throws:**: `ELException`

---


### setValue

```
void setValue(EvaluationContext ctx,
              java.lang.Object value)
              throws ELException
```

:   **Throws:**: `ELException`

---


### getType

```
java.lang.Class getType(EvaluationContext ctx)
                        throws ELException
```

:   **Throws:**: `ELException`

---


### isReadOnly

```
boolean isReadOnly(EvaluationContext ctx)
                   throws ELException
```

:   **Throws:**: `ELException`

---


### accept

```
void accept(NodeVisitor visitor)
            throws ELException
```

:   **Throws:**: `ELException`

---


### getMethodInfo

```
MethodInfo getMethodInfo(EvaluationContext ctx,
                         java.lang.Class[] paramTypes)
                         throws ELException
```

:   **Throws:**: `ELException`

---


### invoke

```
java.lang.Object invoke(EvaluationContext ctx,
                        java.lang.Class[] paramTypes,
                        java.lang.Object[] paramValues)
                        throws ELException
```

:   **Throws:**: `ELException`


---


|  |  |  |
| --- | --- | --- |
| **Overview** \| **Package** \| **Class** \| **Use** \| **Tree** \| **Deprecated** \| **Index** \| **Help** | |  |
| **PREV CLASS**   **NEXT CLASS** | **FRAMES**    **NO FRAMES**     **All Classes** |  |
| SUMMARY: NESTED \| FIELD \| CONSTR \| METHOD | DETAIL: FIELD \| CONSTR \| METHOD |  |


---
